# The complete genome sequence of EC1-UPM, a novel N4-like bacteriophage that infects *Escherichia coli* O78:K80

**DOI:** 10.1186/1743-422X-10-308

**Published:** 2013-10-18

**Authors:** Han Ming Gan, Chin Chin Sieo, Shirley Gee Hoon Tang, Abdul Rahman Omar, Yin Wan Ho

**Affiliations:** 1 Laboratory of Vaccines and Immunotherapeutics, Institute of Bioscience, Universiti Putra Malaysia, 43400 UPM Serdang, Selangor, Malaysia; 2 School of Science, Monash University Malaysia, Bandar Sunway, Malaysia; 3 Department of Microbiology, Faculty of Biotechnology and Biomolecular Sciences, Universiti Putra Malaysia, 43400 UPM Serdang, Selangor, Malaysia; 4 Department of Pathology and Veterinary Microbiology, Faculty of Veterinary Medicine, Universiti Putra Malaysia, 43400 UPM Serdang, Selangor, Malaysia

**Keywords:** Bacteriophage EC1-UPM, Tail fiber protein, Complete genome, Multilocus phylogenetic analysis

## Abstract

**Background:**

Bacteriophage EC1-UPM is an N4-like bacteriophage which specifically infects *Escherichia coli* O78:K80, an avian pathogenic strain that causes colibacillosis in poultry. The complete genome sequence of bacteriophage EC1-UPM was analysed and compared with other closely related N4-like phage groups to assess their genetic similarities and differences.

**Results:**

Bacteriophage EC1-UPM displays a very similar codon usage profile with its host and does not contain any tRNA gene. Comparative genomics analysis reveals close resemblance of bacteriophage EC1-UPM to three N4-like bacteriophages namely vB_EcoP_G7C, IME11 and KBNP21 with a total of 44 protein coding genes shared at 70% identity threshold. The genomic region coding for the tail fiber protein was found to be unique in bacteriophage EC1-UPM. Further annotation of the tail fiber protein using HHpred, a highly sensitive homology detection tool, reveals the presence of protein structure homologous to various polysaccharide processing proteins in its C-terminus. Leveraging on the availability of multiple N4-like bacteriophage genome sequences, the core genes of N4-like bacteriophages were identified and used to perform a multilocus phylogenetic analysis which enabled the construction of a phylogenetic tree with higher confidence than phylogenetic trees based on single genes.

**Conclusion:**

We report for the first time the complete genome sequence of a N4-like bacteriophage which is lytic against avian pathogenic *Escherichia coli* O78:K80. A novel 928 amino acid residues tail fiber protein was identified in EC1-UPM which may be useful to further the understanding of phage-host specificity. Multilocus phylogenetic analysis using core genes of sequenced N4-like phages showed that the evolutionary relationship correlated well with the pattern of host specificity.

## Background

*Escherichia coli* O78:K80 is one of the common serogroups of Avian Pathogenic *Escherichia coli* (APEC) which causes colibacillosis in all ages of chickens, turkeys and other avian species. The infection which is associated with respiratory infection (airsacculitis), followed by perihepatitis, pericarditis and septicaemia is a devastating infection as it may reduce growth and egg production of poultry. Under untreated condition, high mortality rates of birds are recorded and this incurs high economic losses to farmers and the poultry industry [1]. These bacteria enter the human food chain through contamination of the environment by fecal droppings from infected chickens and soiled poultry products [2]. Conventional treatment by using antibiotics has been reported less effective in recent years due to the emergence of antibiotic resistance in the causative agent [3].

In our previous studies, bacteriophage EC1-UPM which was isolated from chicken faecal sample is able to reduce the severity of infection caused by *E. coli* O78:K80 and has the potential to be used for the treatment of colibacillosis in chickens. Based on the results of our *in vivo* study, the total mortality rate of the chickens was reduced by 70% when infected chickens were treated with bacteriophage EC1-UPM. The body weights of treated chickens were 15.4% higher than those of the untreated chickens [4,5]. To further exploit the potential of bacteriophage EC1-UPM, it is essential to have an understanding on its genetic make-up, particularly the genes which are responsible for the infection and lysis of the host bacteria.

In this study, we report for the first time the genome of a bacteriophage EC1-UPM, an N4-like bacteriophage that infects *E. coli* O78:K80. The genetic components of bacteriophage EC1-UPM which may be responsible for its host specificity and virulence were identified and analyzed *in-silico*. We also identified the core genes from the available N4-like bacteriophage genomes and utilized this information to gain insights into the evolutionary relationship of N4-like bacteriophages using multilocus phylogenetic analysis.

## Results and discussion

### Genomic features of bacteriophage EC1-UPM

**Figure 1:**
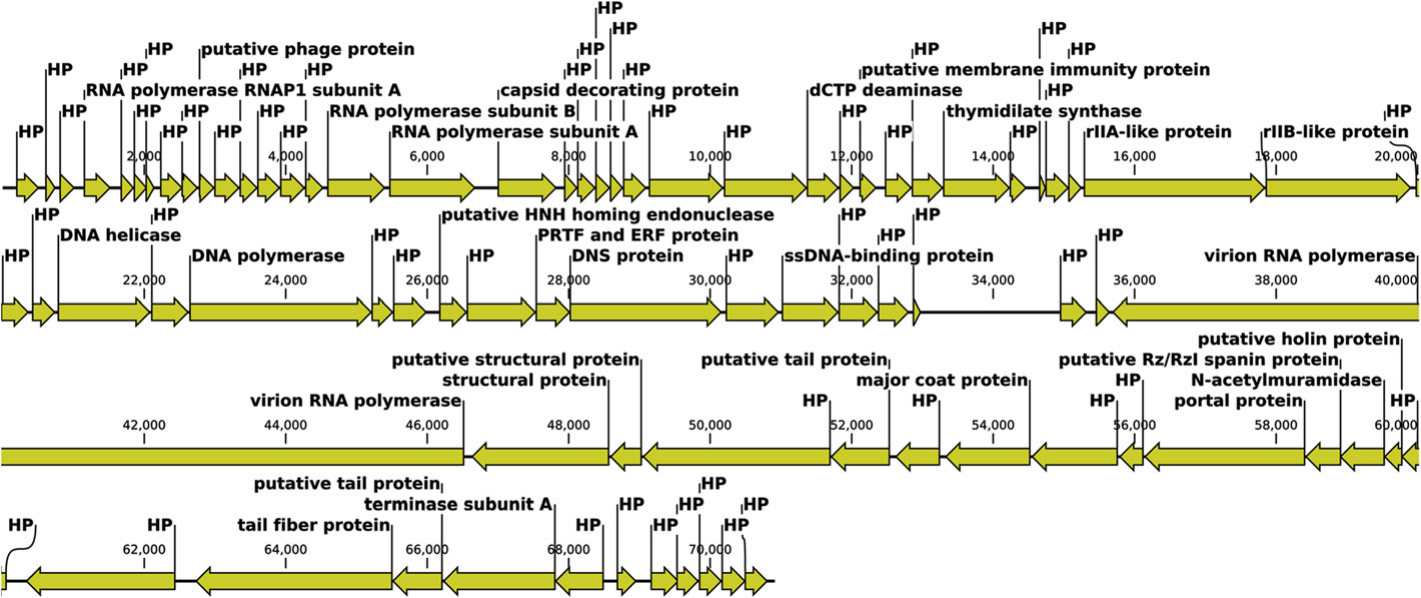
**Linear genome visualization of bacteriophage EC1-UPM. Direction of the arrow represents transcription orientation.** HP, hypothetical protein; PTF, pathogenesis-related transcription factor; DNS, extracellular deoxyribonuclease.

The genome of bacteriophage EC1-UPM consists of 70,912 bp (GC content of 42.9%). A total of 80 protein coding genes were predicted with an average length of 816 bp (92.5% coding density). Annotation of the majority of predicted CDS showed confident hits against proteins of the N4-like bacteriophages, indicating that bacteriophage EC1-UPM may be a member of the N4-like bacteriophages (Additional file 1: Table S1). N4-like bacteriophages are bacteriophages of the *Podoviridae* family. Members of this group are lytic against their hosts. The host specificity of N4-like bacteriophages is rather diverse, ranging from enterobacteria such as *E. coli* and *Pseudomonas aeruginosa* to marine bacteria such as *Sulfitobacter* sp. EE-36 and *Silicibacter pomeroyi* DSS-3 [6-9]. Bacteriophage N4 which infects *E. coli* K-12 is currently the most studied strain for this group of bacteriophages [10]. In addition to having its complete genome sequenced, the identity and locations of several of its structural proteins have been established through comparisons of three-dimensional, cryo-electron microscopic structures of wild-type N4 and its mutants [11]. Based on our analysis, the annotated proteins of bacteriophage EC1-UPM can be categorized into the following functional groups: bacteriophage structure and packaging (portal protein, major coat protein, tail protein, tailspike protein, structural protein, capsid decorating protein), DNA replication/modification (DNA helicase, DNA polymerase, endonuclease, terminase), signal transduction and regulatory function (ssDNA-binding protein, RNA polymerase), nucleotide metabolism (thymidilate synthase, dCTP deaminase) and host lysis (holin, N-acetylmuramidase). The presence of lysis gene but not lysogeny-related gene indicates that bacteriophage EC1-UPM is a lytic bacteriophage. The largest gene encodes for the virion polymerase and is 10,839 bp, which is approximately 15% of the whole genome length (Figure 1). The presence of bacteriophage-encoded RNA polymerase of such size is a signature of N4 bacteriophage. It was demonstrated in N4 bacteriophage that this particularly large RNA polymerase was packaged into its capsid and ejected into the host cell thus eliminating the need to rely on host RNA polymerase for the transcription of its early genes [11]. No tRNA was identified in the genome of bacteriophage EC1-UPM, thus indicating that upon entry into the host, it is completely reliant on the host tRNA makeup for its protein synthesis.

### Codon usage of bacteriophage EC1-UPM

**Figure 2:**
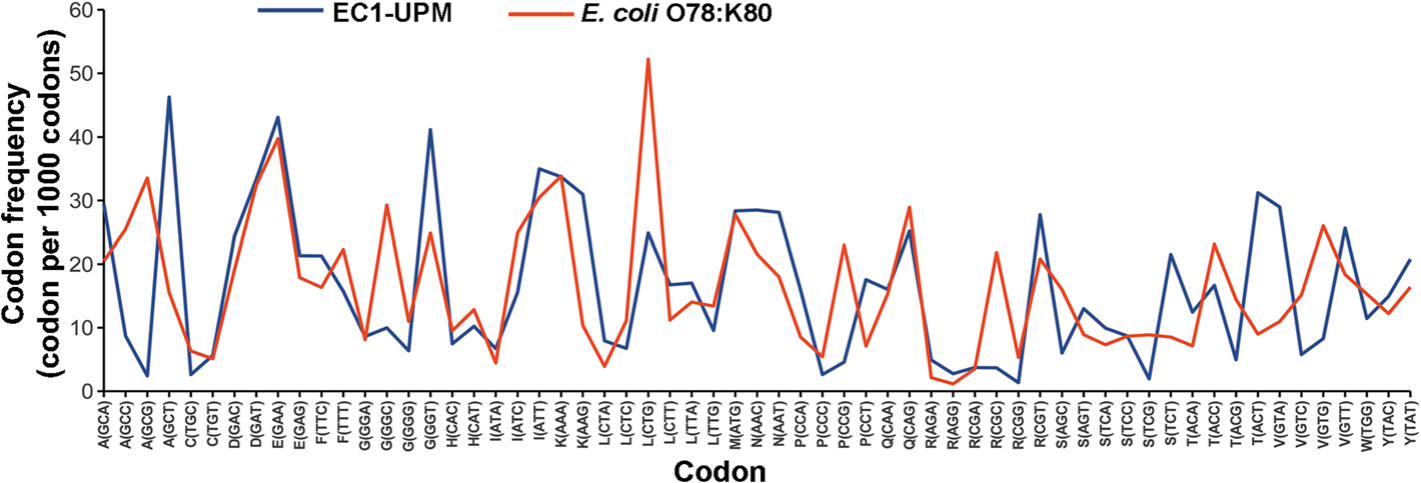
**Codon frequency of bacteriophage EC1-UPM and its host *E. coli *O78:K80. Non-bracketed letters on the X-axis represent the amino acid associated with the codon.**

Comparison of the codon usage in *E. coli* O78:K80 and bacteriophage EC1-UPM revealed a rather similar codon frequency (Figure 2). Compatibility of phage codon-usage with that of its host will confer an evolutionary advantage as this will aid in the energy-demanding and laborious mechanism of protein synthesis. Three codons however were overrepresented in bacteriophage EC1-UPM, namely, codons ACT, GTA and GCT that correspond to the amino acids threonine, valine and alanine, respectively. Interestingly, bacteriophage EC1-UPM does not adapt to the codon usage dissimilarity as evidence by the absence of tRNA gene in its genome. In the evolution of dsDNA phages, the presence of tRNAs particularly those that are abundant in the phage and less common in the host allows the phage to outcompete its potential competitors in the host by translating its proteins more effectively, speeding up the reproduction rate and reducing its latency time [12]. In T4 phages, deletion of the tRNA genes was shown to decrease burst sizes and protein synthesis rates, demonstrating the growth advantage of carrying tRNA genes in phages [13]. The complete absence of tRNA in bacteriophage EC1-UPM may imply that its ability to infect *E. coli* O78:K80 could have been a recent acquisition.

### Comparative genomics of bacteriophage EC1-UPM and closely related N4-like bacteriophages

**Figure 3:**
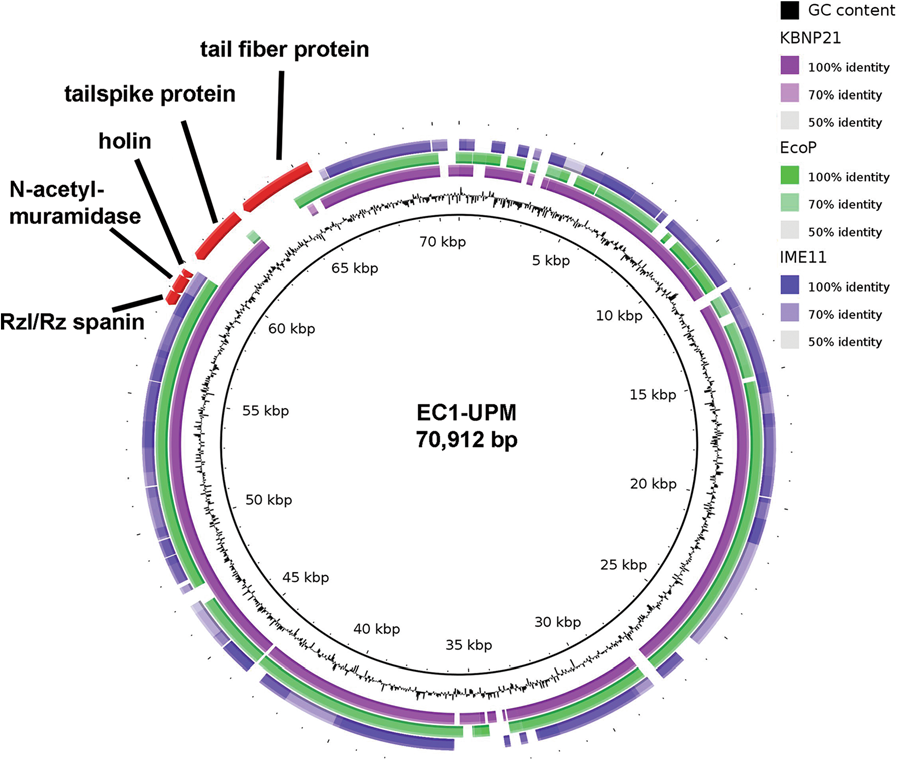
**Blast ring image using the complete genome of bacteriophage EC1-UPM as reference.** Outer rings represent the genomes of three N4-like bacteriophages closely related to EC1-UPM.

**Figure 4:**
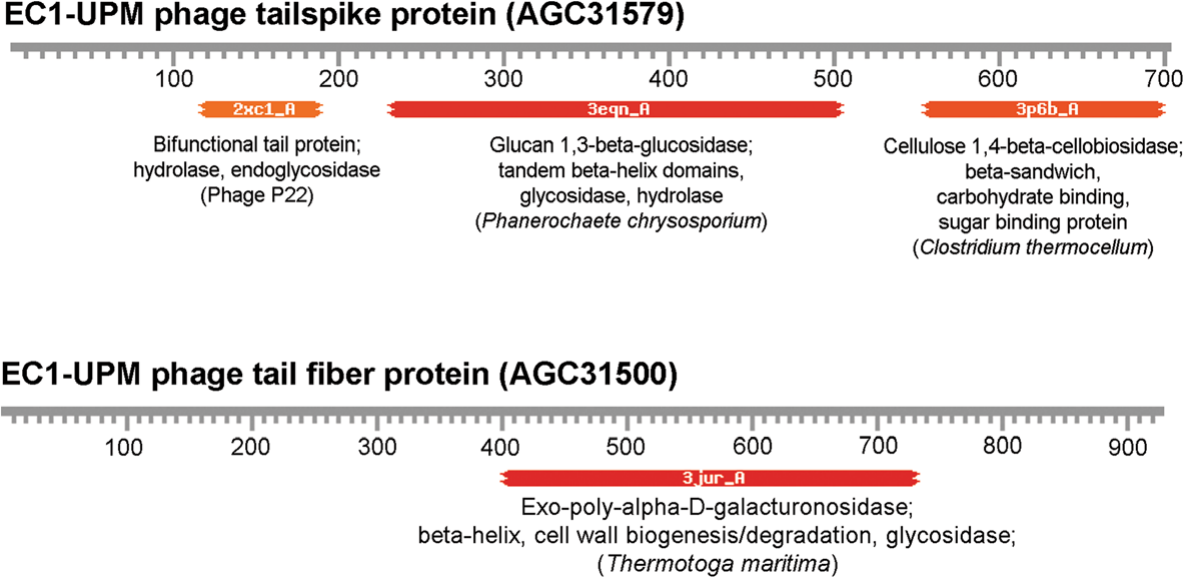
**Identification of protein regions homologous to polysaccharide degrading enzymes in the tailspike and tail fiber proteins of bacteriophage EC1-UPM via HHpred.** Protein IDs are indicated by the bracketed text.

Initial whole genome BLAST of bacteriophage EC1-UPM against the NCBI database showed that bacteriophage EC1-UPM is closely related to three N4-like viruses namely KBNP21, vB_EcoP_G7C and IME11 (data not shown). Bacteriophages KBNP21, vB_EcoP_G7C and IME11 were isolated from chicken farm, horse feces and hospital sewage, respectively [6,14,15]. Bacteriophage IME11 could infect 13 of 31 enteropathogenic clinical *E. coli* strains [6]. Bacteriophage vB_EcoP_G7C could infect *E. coli* strain 4 while bacteriophage KBNP21 could infect *E. coli* strains KBP21 and KBP135 [14,15]. However, the lack of O and K antigen data of the tested *E. coli* strains severely impeded host specificity determination based on surface antigen. Using Blast Ring Image generator (BRIG), it was shown that the majority of genomes are conserved across the four bacteriophages (Figure 3), except in the genomic region encompassing the genes coding for tail fiber and tail spike proteins. Tail fiber and tail spike proteins are often associated with the binding and degradation of host cell surface antigen [14]. Given the differences in the isolation source of each *E. coli*-infecting N4-like bacteriophage, it is likely that their *E. coli* hosts are of different strains that lack similarity in surface antigen profile. The gene coding for tail spike protein in bacteriophage EC1-UPM is conserved only in bacteriophage KBNP21 which was similarly isolated from avian source. Although, InterProscan annotation of the tail spike protein did not show any significant protein domain (Additional file 1: Table S1), augmentation of the protein annotation with HHpred indicates three major domains associated with carbohydrate binding and degradation between residues 120 to 190, 230 to 500 and 550 to 700 (Figure 4). Tail spike has been shown to be a vital component particularly in the infection of bacteria possessing both O and K antigens [14]. Some K antigens could prevent the direct recognition of receptor on the O antigen by the phage tail due to the large K-antigen size (up to 4000 A). The tail spike protein functions to overcome such limitation by creating a tunnel in the cell capsule and exposing the receptor to the tail protein [15,16].

### Novel region in the C-terminus of tail fiber protein

Based on BRIG visualization, the genomic region corresponding to the C-terminus of bacteriophage EC1-UPM tail fiber protein was not shared by any member of the closely related N4-like bacteriophages (Figure 3). Similar to tail spike protein, the tail fiber of bacteriophage has also been implicated in host specificity. The C-terminus region of tail fiber protein has been reported to alter the host range of a bacteriophage [17]. Divergences in the C-terminus may therefore represent the phage adaptation towards the huge variation of O and K antigens in *E. coli*[18]. Expanding the BLAST search towards the whole NR database did not reveal any significant hit in the C-terminus region. Based on HHpred annotation, the C-terminus of tail fiber protein showed significant degree of structural homology with various polysaccharide degrading enzymes possessing the right-handed β-helix structure (Figure 4 and Additional file 2: Table S2). Notably, residues 402 to 731 of the tail fiber displayed significant similarity to a β-helix structure-containing exo-poly-α-D-galacturonosidase of *Thermotoga maritima,* a thermophilic bacteria (E-value = 4.44 × 10^-44^) (Figure 4 and Additional file 2: Table S2). Exo-poly-α-D-galacturonosidase is involved in the hydrolysis of pectic from the non-reducing to release digalacturonate. Initially identified in pectate lyase C, the right-handed β-helix is commonly found in the catalytic domains of various proteins that process oligo- and polysaccharides [19]. Given the structural homology of the tail fiber C-terminus to various polysaccharide degrading enzymes (Additional file 2: Table S2), it is plausible that the tail fiber may be associated with binding and/or degradation of the polysaccharide present on the bacterial antigens. The presence of hydrolytic phage tail fiber proteins has been shown to allow bacteriophage ΦK1-5 to attach and degrade both K1 and K5 capsular polysaccharides [20]. Further enzymatic study is necessary to validate the function of bacteriophage EC1-UPM tail fiber protein and its substrate range. This novel tail fiber protein consisted of 928 amino acid residues with molecular mass and pI of 99.3 kDa and 4.85, respectively.

### Host lysis

It is widely accepted that lysis of the host is indispensable for the release and dispersion of progenies in all bacteriophages, except in filamentous bacteriophages [21,22]. In bacteriophage EC1-UPM, genes involved in the holin-endolysin system are located adjacent to each other. The N-acetylmuramidase (NCBI accession number: AGC31576) which may be responsible for the endolysis activity contains a domain implicated in glycosyl hydrolase (PF05838) from residues 22 to 113. The glycosyl hydrolase family functions via the hydrolysis of β-1,4 bond between N-acetylmuramic acid and N-acetylglucosamine in the peptidoglycan layer of bacteria. The presence of a peptidoglycan binding domain (PF09374) from residue 116 to 195 after the glycosyl hydrolase domain may further enhance the activity of N-acetylmuramidase by localizing the protein to its target region. Membrane topology visualization of bacteriophage EC1-UPM holin protein indicates that it contains two transmembrane domains with both N-and C-terminus (data not shown) located within the cytoplasmic region, thus classifying it as a class II holin [21,22]. The genes coding for the holin-endolysin system are well-conserved in bacteriophages KBNP21, vB_EcoP_G7C, IME11 and EC1-UPM (Figure 3), indicating a common mechanism for host lysis.

### Orthologs identification and multilocus phylogenetic analysis of N4-like bacteriophages

**Figure 5:**
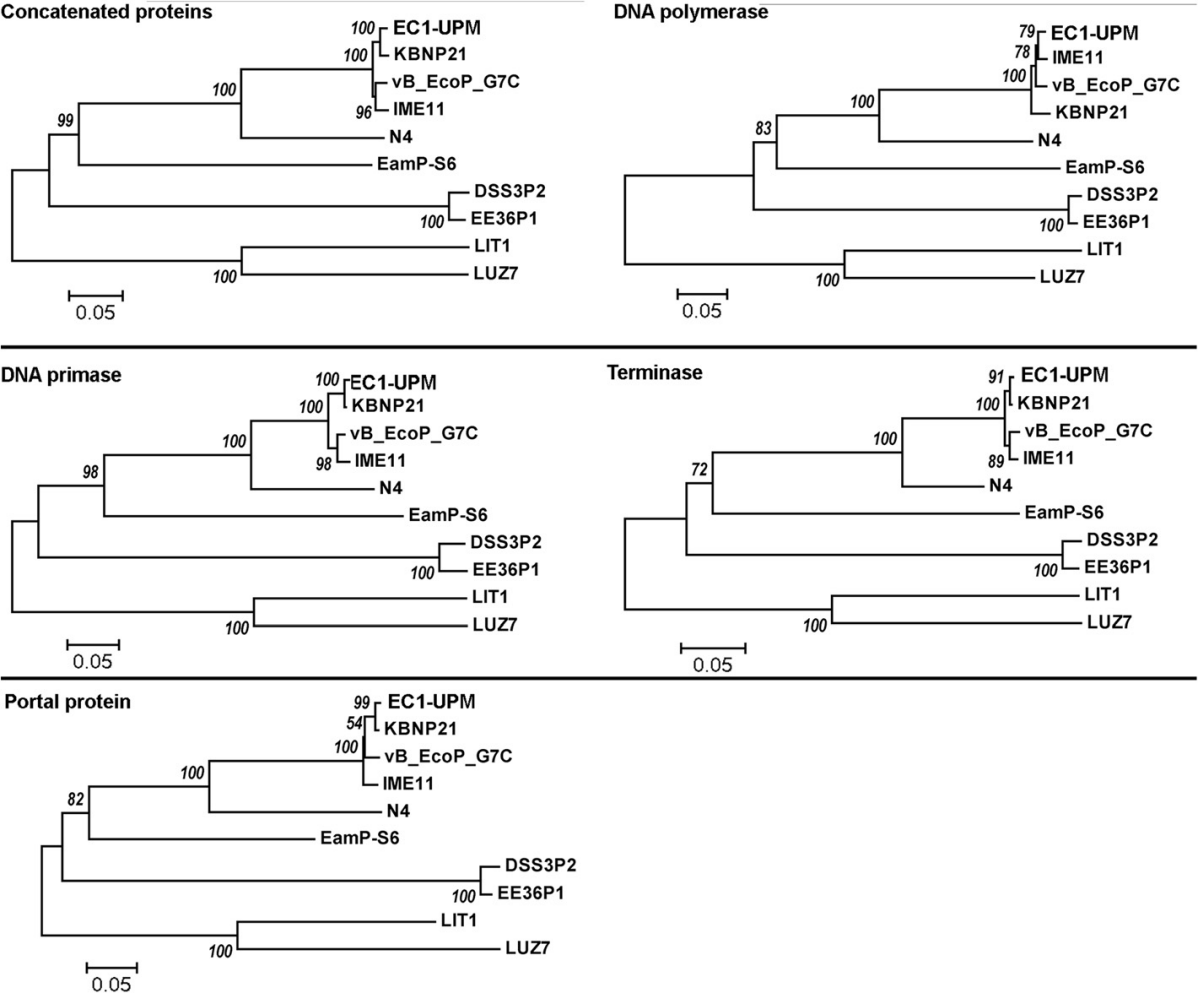
**Elucidation of the evolutionary relationship of various N4-like bacteriophages based on multilocus and single-gene phylogenetic analyses.** A total of seven orthologous groups were used for the multilocus phylogenetic analysis. Four out of the seven orthologous groups contained functional assignment and were subsequently chosen for the construction of an individual single-gene phylogenetic tree.

The recent steady increase and availability of the complete genomes of various N4-like bacteriophages in the GenBank provides an opportunity to identify the orthologs that are well-conserved in all N4-like bacteriophages. Initial orthologs identification in bacteriophage EC1-UPM and its closely related strains vB_EcoP_G7C, IME11 and KBNP21 revealed that at 70% identity and 70% aligned cutoffs, a total of 44 proteins were conserved across the bacteriophages (Table 1). The average identities of the orthologs shared in bacteriophages vB_EcoP_G7C, IME11 and KBNP21 as compared to that of EC1-UPM were 91%, 93% and 95%, respectively. No orthologs can be identified when the analysis was expanded to include the more distant N4-like bacteriophages (Table 2) using the similar setting. Nevertheless, by lowering the identity cutoff to 40%, seven orthologous clusters were identified namely DNA polymerase I, N4 gp42-like protein, DNA primase, N4 gp44-like protein, portal protein, terminase and N4 gp69-like protein. The functional assignment of proteins in the orthologous clusters is in agreement with the notion that viruses share homologous “hallmark genes” encoding for proteins involved in genome replication and virion structure formation [23]. The phylogenetic tree analysis utilizing the trimmed and concatenated alignment of proteins in these orthologous clusters showed that the evolutionary relationship of the N4-like bacteriophage is very much correlated with their host specificity. Comparison of several phylogenetic trees constructed using individual orthologous clusters with known function (DNA polymerase, terminase, portal protein and DNA primase) showed that phylogenetic tree analysis based solely on sequence alignment of DNA polymerase resulted in a slightly different tree topology with a lower bootstrap support as compared to the multi-locus phylogenetic tree (Figure 5). This demonstrates that although DNA polymerase is a good indicator of evolutionary relationship in general, caution should be taken when resolving closely related N4-like bacteriophages. In contrast, phylogenetic tree construction based on DNA primase is highly comparable with the multi-locus phylogeny approach in terms of tree topology and bootstrap support value.

**Table 1:** Orthologous group of proteins conserved across bacteriophages EC1-UPM, vB_EcoP_G7C (E), IME11 (I) and KBNP21 (K) as identified by PanOCT (>70% Identity and > 70% aligned length)

**Protein ID**	**Product Description**	**% Identity**			**InterProscan ID**
		**E**	**I**	**K**	
AGC31510	hypothetical protein	87	95	97	
AGC31513	RNA polymerase RNAP1 subunit A	96	94	98	
AGC31517	hypothetical protein	94	93	96	
AGC31519	putative phage protein	74	90	84	
AGC31522	hypothetical protein	82	99	99	
AGC31523	hypothetical protein	91	88	91	
AGC31525	RNA polymerase subunit B	95	96	99	
AGC31526	RNA polymerase subunit A	88	96	97	IPR002092
AGC31527	capsid decorating protein	79	91	93	IPR003599; IPR007110; IPR013783
AGC31528	hypothetical protein	98	97	97	
AGC31531	hypothetical protein	95	97	95	
AGC31533	hypothetical protein	95	95	95	IPR027417
AGC31534	hypothetical protein	95	97	99	IPR018698; IPR025154
AGC31537	putative membrane immunity protein	86	98	94	IPR016410
AGC31539	hypothetical protein	88	94	95	
AGC31540	thymidilate synthase	84	90	95	IPR003669
AGC31543	hypothetical protein	76	80	82	
AGC31545	rIIA-like protein	86	91	94	IPR003594
AGC31546	rIIB-like protein	88	89	87	
AGC31547	hypothetical protein	90	96	95	
AGC31549	DNA helicase	98	99	99	IPR027417; IPR027785
AGC31550	hypothetical protein	97	99	98	
AGC31551	DNA polymerase	98	98	97	IPR001098; IPR002298; IPR012337
AGC31552	hypothetical protein	92	91	99	
AGC31555	hypothetical protein	97	95	98	
AGC31557	DNS protein	97	96	100	IPR014820
AGC31558	hypothetical protein	99	99	99	
AGC31559	ssDNA-binding protein	95	97	97	
AGC31560	hypothetical protein	99	99	98	
AGC31561	hypothetical protein	76	92	94	
AGC31567	putative structural protein	88	85	85	
AGC31568	hypothetical protein	92	93	94	
AGC31569	putative tail protein	94	82	96	
AGC31570	hypothetical protein	96	97	99	
AGC31571	major coat protein	97	97	100	
AGC31572	hypothetical protein	95	93	96	
AGC31573	hypothetical protein	99	96	100	
AGC31574	portal protein	97	97	99	
AGC31576	N-acetylmuramidase	95	97	98	IPR008565; IPR018537
AGC31502	terminase subunit A	98	98	100	IPR004921
AGC31503	hypothetical protein	98	98	100	
AGC31504	hypothetical protein	80	91	85	
AGC31505	hypothetical protein	81	76	77	
AGC31506	hypothetical protein	87	74	80	

**Table 2:** General features and genome accession numbers of the N4-like bacteriophages involved in the multilocus phylogenetic study

**Phage**	**Isolation source**	**Host**	**Accession number**	**Reference**
EC-UPM	Chicken fecal material	*E. coli*	KC206276	This study
KBNP21	Chicken farm	*E. coli*	JX415535	[24]
vB_EcoP_G7C	Horse fecal material	*E. coli*	HQ259105	[25]
IME11	Hospital sewage	*E. coli*	JX880034	[6]
N4	Sewer	*E. coli*	EF056008	[11]
EamP-S6	Fruit production environment	*Erwinia amylovora*	NC_019514	[17]
DSS3P2	Seawater sample	*Silicibacter pomenyi*	Nc_012697	[7]
EE36P1	Seawater sample	*Sulfitobacter sp.*	NC_012696	[7]
LIT1	Pond	*Pseudomonas aeruginosa*	NC_013692	[9]
LUZ7	Hospital waste sample	*Pseudomonas aeruginosa*	NC_013691	[9]

## Conclusions

We reported for the first time the genome sequence of a N4-like bacteriophage which is capable of infecting the avian pathogenic *Escherichia coli* O78:K80. The analysis of its genome identified a novel genomic region which may be responsible for its host specificity. This study also identified the orthologous groups of protein shared by all sequenced N4-like phages and showed that the evolutionary relationship of N4-like bacteriophages correlated with the pattern of host specificity.

## Material and methods

### Genome sequencing and annotation of bacteriophage EC1-UPM

Methods for propagation, purification and genomic DNA extraction of bacteriophage EC1-UPM followed those described by Lau et al. [4]. The purified DNA was sequenced on the Illumina Hiseq 2000 (100-bp paired-end reads). The reads were assembled *de novo* using Velvet v1.1.07. Subsequent scaffolding and gap filling were performed to obtain a complete genome of bacteriophage EC1-UPM. Protein coding sequences were predicted using combined gene model from GeneMarkS and Glimmer3 [26]. The predicted proteins were annotated by BLAST against the NCBI non-redundant database. Additional annotation was subsequently performed using Blast2Go and InterProscan [27,28]. Possible presence of tRNA in the genome was determined using tRNAscan-SE v1.23.

### Genome visualization of bacteriophage EC1-UPM and comparison with other closely related bacteriophage genomes

The annotated complete genome of bacteriophage EC1-UPM was visualized using CLC Genomics Workbench 6.0 (CLC Bio, Denmark). To identify the closely related bacteriophage, BLASTN was performed on the complete genome of bacteriophage EC1-UPM against the NCBI non-redundant database. Blast Ring Image Generator [29] was used to provide the genomic comparison of bacteriophage EC1-UPM against its closely related bacteriophage (40% identity cut-off).

### *In-silico* analysis of bacteriophage EC1-UPM tailspike and tail fiber proteins

Additional annotation of the tail fiber protein was performed using the HHpred interactive server [30]. Additionally, the computation of various physical and chemical parameters of the tail fiber protein was performed using ProtParam [31].

### Classification of bacteriophage EC1-UPM holin

Protein sequence of the putative holin was subjected to a transmembrane helices prediction using the TMHMM Server v. 2.0. The text output from the prediction was subsequently piped into to TMRPres2D [32] to create uniform, two-dimensional high analysis graphical images of the putative transmembrane protein.

### Evolutionary relationship of the N4-like bacteriophages

The currently available genomes of N4-like bacteriophage were retrieved from NCBI in Genbank format. PanOCT [33] was used to identify the genes that are conserved across the N4-like bacteriophages (40% or 70% identity cut-off, at least 70% protein length overlap). Each orthologs cluster was aligned using MUSCLE [34]. Then, the individual alignment was trimmed with TrimAl using the “strictplus” parameter which is optimized for NeighborJoining tree construction [35] and concatenated for phylogenetic analysis using MEGA. A phylogenetic tree was constructed using the NeighborJoining method with 1000 bootstrap repetitions.

### Nucleotide sequence accession number

The Genbank accession number for the complete genome sequence of bacteriophage EC1-UPM is KC206276.

## Competing interests

The authors declare that they have no competing interests.

## Authors’ contributions

HMG carried out genetic analysis, drafted the manuscript. CCS designed the whole process and helped editing the manuscript. SGHT participated in the experiments. ARO and YWH provided the facilities and reviewed the manuscript. All authors read and approved the final manuscript.

## Supplementary Material

Additional file 1: Table S1 Blast2Go and InterProscan annotation of all predicted proteins in bacteriophage EC1-UPM. Click here for file

Additional file 2: Table S2 HHpred annotation of the tail fiber protein of bacteriophage EC1-UPM. Click here for file
